# Thermal Recovery of the Electrochemically Degraded LiCoO_2_/Li_7_La_3_Zr_2_O_12_:Al,Ta Interface in an All-Solid-State Lithium Battery

**DOI:** 10.1021/acsami.2c20004

**Published:** 2023-01-17

**Authors:** Martin Ihrig, Liang-Yin Kuo, Sandra Lobe, Alexander M. Laptev, Che-an Lin, Chia-hao Tu, Ruijie Ye, Payam Kaghazchi, Luca Cressa, Santhana Eswara, Shih-kang Lin, Olivier Guillon, Dina Fattakhova-Rohlfing, Martin Finsterbusch

**Affiliations:** † Institute of Energy and Climate Research − Materials Synthesis and Processing, Forschungszentrum Jülich GmbH, 52425Jülich, Germany; ‡ Department of Chemical Engineering, Ming Chi University of Technology, No. 84, Gungjuan Road, New Taipei City24301, Taiwan; § Łukasiewicz Research Network − Poznan Institute of Technology, 6 Ewarysta Estkowskiego St., 61-755Poznań, Poland; ∥ Department of Materials Science and Engineering, National Cheng Kung University, No. 1, University Road, Tainan City701, Taiwan; ⊥ Hierarchical Green-Energy Materials Research Center, National Cheng Kung University, No. 1, University Road, Tainan City701, Taiwan; # MESA+ Institute for Nanotechnology, University of Twente, P.O. Box 217, Enschede7500AE, The Netherlands; ● Luxembourg Institute of Science and Technology, Advanced Instrumentation for Nano-Analytics (AINA), rue du Brill 41, 4422Belvaux, Luxembourg; □ Program on Smart and Sustainable Manufacturing, Academy of Innovative Semiconductor and Sustainable Manufacturing, National Cheng Kung University, Tainan City701, Taiwan; ∇ Jülich-Aachen Research Alliance: JARA-ENERGY, 52425Jülich, Germany; ◇ Faculty of Engineering and Center for Nanointegration Duisburg-Essen, University Duisburg-Essen, Lotharstr. 1, 47057Duisburg, Germany

**Keywords:** ASSLB, solid-state Li battery, cathode/garnet interface, interface degradation, thermal treatment, interface recovery

## Abstract

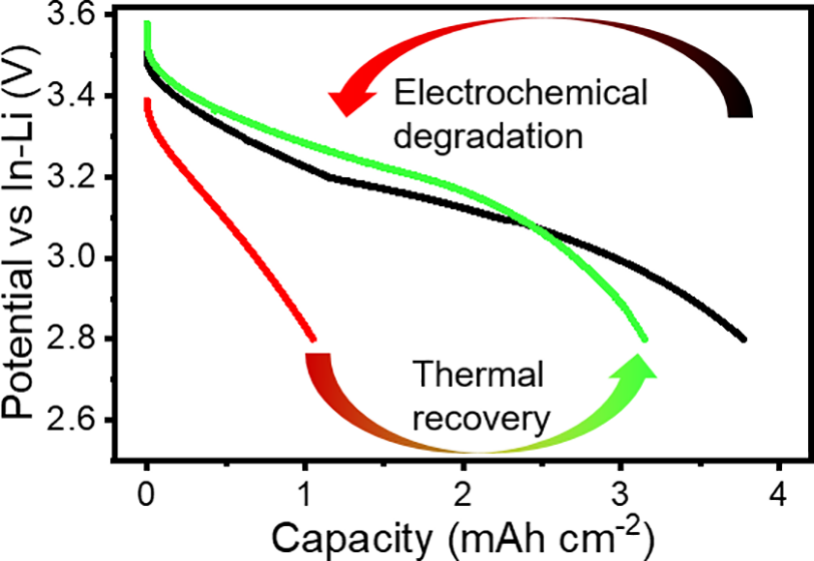

All-solid-state lithium batteries are promising candidates for next-generation energy storage systems. Their performance critically depends on the capacity and cycling stability of the cathodic layer. Cells with a garnet Li_7_La_3_Zr_2_O_12_ (LLZO) electrolyte can show high areal storage capacity. However, they commonly suffer from performance degradation during cycling. For fully inorganic cells based on LiCoO_2_ (LCO) as cathode active material and LLZO, the electrochemically induced interface amorphization has been identified as an origin of the performance degradation. This study shows that the amorphized interface can be recrystallized by thermal recovery (annealing) with nearly full restoration of the cell performance. The structural and chemical changes at the LCO/LLZO heterointerface associated with degradation and recovery were analyzed in detail and justified by thermodynamic modeling. Based on this comprehensive understanding, this work demonstrates a facile way to recover more than 80% of the initial storage capacity through a thermal recovery (annealing) step. The thermal recovery can be potentially used for cost-efficient recycling of ceramic all-solid-state batteries.

## Introduction

All-solid-state lithium batteries (ASSLBs) based on Li-ion conducting oxide ceramics have intrinsic safety and an extended operational temperature range and show the potential to achieve high energy density. These properties make them a candidate for the next generation of ASSLBs.^1−8^ One of the most promising solid electrolytes is the Li_7_La_3_Zr_2_O_12_ (LLZO) oxide substituted with Al and Ta. This solid electrolyte shows a high ionic conductivity of up to 1 mS cm^–1^ at room temperature and chemical stability against the Li anode.^2,3,9−16^ However, the fabrication of ASSLBs based on LLZO is challenging due to the interaction of cathode active materials (CAMs) and LLZO at elevated temperatures.^3,11,12,14,17^ A high sintering temperature range of 1000 °C to 1200 °C is required to obtain a well-conducting LLZO network. Typically this temperature range exceeds the thermal stability of LLZO in contact with high-capacity or high-voltage CAMs such as LiNi_0.33_Co_0.33_Mn_0.33_O_2_ or LiMn_1.5_Ni_0.5_O_4_ due to reaction caused by thermal instability above 600 °C.^12,16,18,19^ So far, only LiCoO_2_ (LCO) in combination with LLZO shows sufficiently high thermal stability until 1075 °C,^20^ opening a process window for fabrication of fully ceramic solid-state (LCO-LLZO:Al,Ta)-based Li batteries.^2,5,9,17,21,22^

The fabrication of ASSLBs with LCO and LLZO by various processing techniques has been demonstrated in the literature.^2,5,9,17,21,22^ However, the cells either suffer from rapid performance degradation or show low storage capacity.^2,5,9,17,21,22^ Several groups proposed degradation mechanisms, such as chemical instability during processing, fracture of rigid solid–solid interfaces, or electrochemical decomposition.^2,9,10,23^ However, a detailed and comparable analysis of the degradation mechanism in cells is difficult to perform because of various cell designs, cell characteristics, and manufacturing routes. Most likely, a combination of different degradation mechanisms occurs.

In general, there are three main degradation mechanisms affecting the cell performance. The first degradation mechanism originates from the chemical instability of the CAM and/or the solid electrolyte during sintering at elevated temperatures. Such instabilities have been described by several groups and always lead to the formation of a secondary phase that renders the battery inoperable. Even in the case of LCO/LLZO, some diffusion across the interface leads to the formation of secondary phases with low conductivity, such as the Li-poor La_2_Zr_2_O_7_ or Li_*x*_La_*y*_Co_*z*_O, which impedes the transfer of Li ions.^13,24−26^ The common strategy to prevent the formation of secondary phase formation is the use of a sintering additive such as Li_3_BO_3_, which, however, has low intrinsic ionic conductivity.^27^ In any case, any addition of sintering additives or surface modifications requires an additional processing step and can lead to new undesired effects.^28−31^ Advanced sintering techniques, in particular field-assisted sintering technique also known as spark plasma sintering (FAST/SPS), can also mitigate the secondary phase formation and results in clean interfaces and good electrochemical performance.^5,17^ The application of mechanical pressure during FAST/SPS enables the manufacturing of dense components for ASSLBs at reduced temperature and shorter dwell time without formation of secondary phases (Figure S1).^32−34^

Once a temperature window ensuring the material stability during sintering is defined, a second degradation mechanism is observed during the electrochemical cycling of cells. This mechanism is especially relevant for composite LCO-LLZO cathodes with residual porosity. The degradation occurs due to fracture of the LCO/LLZO interface initiated by the volume change of LCO. Numerical modeling explains this failure mechanism due to the high stresses in the vicinity of the LCO/LLZO interface.^35^ In particular, an inhomogeneous stress distribution which peaks near the pores was found. Thus, the pores can be an origin of microcracks. In contrast, a more homogeneous stress distribution with the reduced probability of cracking was predicted for a pore-free LCO/LLZO interface.^35^ Recently, we experimentally confirmed this conclusion for dense composite LCO-LLZO cathodes (Figure S2).^32^

Nevertheless, even for dense LCO-LLZO cathodes a rapid capacity degradation during cycling was observed due to an additional degradation mechanism. In our previous work,^32^ we have shown that this electrochemically driven degradation is associated with the changes in crystallinity and with the ion diffusion at and through the LCO/LLZO interface. These changes lead to an increase in cathode impedance and to a rapid degradation of the performance. However, it should be noted that the degradation processes are mainly based on the interdiffusion of ions between two solid phases without any change in the aggregation state. This makes the degradation of oxide-based ASSLBs fundamentally different from other types of Li-ion and solid-state Li batteries, including the polymer- or sulfide-based ASSLBs, where degradation results in material loss due to irreversible chemical reactions (e.g., oxidation or reduction of liquid electrolyte at the electrode interface with formation of a solid electrolyte interface (SEI) or thermal runaway of CAM accompanied by gas evolution).^36^ In these cell types, recycling is accomplished by complete decomposition of the cell until the raw material level.^10,37^ In contrast, we have postulated that for our garnet (LCO-LLZO:Al,Ta)-based ASSLB, a thermal recovery (annealing) step could recrystallize the amorphized interlayer with restoring of the cell performance. As in the original manufacturing process, the thermal recovery (annealing) can be performed at the component level (a cathode and a separator) if a Li anode is used, or even at the full cell level (a cathode, a separator, and an anode) if an all-ceramic cell is used. Complete disassembly of the cell is not required for the recycling/recovery of garnet (LCO-LLZO:Al,Ta)-based ASSLBs, which could result in significant energy savings and make the recycling process industrially feasible and environmentally friendly.

In this work, we prove the postulated concept using an garnet (LCO-LLZO:Al,Ta)-based ASSLB fabricated by FAST/SPS. We tracked the electrochemical degradation during cycling and demonstrated the recovery of the cells by a simple heating procedure. Advanced methods of interface analysis such as transmission electron microscopy (TEM), secondary ion mass spectrometry (SIMS),^38^ and micro-Raman spectroscopy have been employed to gain insights into the macroscopic to the atomic scale. Finally, the experimental observations were validated by thermodynamic-based simulations using density functional theory (DFT).

## Materials and Methods

### Materials Synthesis and Preparation of the Half-Cells

Details on the synthesis of the LLZO:Al,Ta (Li_6.45_Al_0.05_La_3_Zr_1.6_Ta_0.4_O_12_) solid electrolyte and preparation of the LCO-LLZO:Al,Ta mixture can be found elsewhere.^17^ The starting powders and LCO-LLZO:Al,Ta mixture were stored in an Ar-filled glovebox (<0.1 ppm of H_2_O and O_2_).

### Full Cell Fabrication

The detailed description of the full cell fabrication is provided elsewhere.^17,32^ The full cells were fabricated with half-cells consisting of a composite LCO-LLZO:Al,Ta cathode (about 170 μm in thickness with an LCO loading of 49 mg) and an LLZO:Al,Ta separator (with a thickness of about 400 μm).

### Thermal Recovery

The cycled cell was disassembled, and the anode and the Au layers were polished off with sandpaper. The remaining half-cell was placed in a furnace and heated to 600 °C at a rate of 2 K/min and then to 1050 °C at 10 K/min. The temperature was maintained for 30 min and was followed by free cooling to room temperature. Then, the recovered half-cell was assembled with a fresh In-Li anode, as described elsewhere.^32^

### Electrochemical Characterization

The initial and recovered full cells were placed in an 80 °C warm climate chamber VT 4002EMC (Vötsch Industrietechnik). The electrochemical measurements were carried out using a BioLogic VMP-300 Multichannel Potentiostat. Electrochemical impedance spectroscopy (EIS) was performed in a frequency range of 3 MHz to 100 mHz and a perturbation field with an amplitude of 10 mV. The electrochemical impedance spectra were fitted using the ZView software (Scribner). Long-term cycling was performed using constant-current–constant-voltage (CC–CV) charging, where the cells were charged to 3.6 V vs In–Li (i.e., 4.2 V vs Li/Li^+^) with a constant current density of 50 μA cm^–2^ and held at a voltage of 3.6 V vs In–Li to allow the current to drop to 10 μA cm^–2^. The cell discharge was performed with a constant current of 50 μA cm^–2^ until the voltage dropped to 2.8 V vs In–Li.

### Microstructural Analysis

The lamella for TEM were cut out of a polished cross section by a dual beam-focused Ga-ion beam with 30 keV and 10 pA within a Helios NanoLab G3 CX device and transferred to a lacey carbon TEM grid by a glass tip micromanipulator. The TEM images were recorded with a JEM-2100F electron microscope (JEOL) operated at 200 kV.

Selected area electron diffraction (SAED) patterns were collected by parallel incident electron beam with a diameter of a few micrometers. The diffracted area of the specimen was selected with the selected-area aperture which is located in the image plane of the objective lens. Thus, the SAED patterns can be collected selectively from the LCO and LLZO:Al,Ta part, and the LCO/LLZO:Al,Ta interfaces.

### Raman Spectroscopy

Raman spectroscopy was carried out with a Renishaw inVia Qontor Raman Microscope equipped with a solid-state 532 nm excitation laser and a 2400 l mm^–1^ grating. The output laser power was limited to about 2.5 mW to avoid material degradation. Both materials, LCO and LLZO:Al,Ta, decompose during exposure to high laser intensities.^26,39,40^ For each sample, an area of 60 μm × 40 μm with a step size of 0.22 μm was mapped, leading to 49051 measurement points per sample.

#### Statistical Raman Spectroscopy Analysis

For data analysis, the cosmic rays were removed by using the implemented method in the Wire 5.2 software package (Renishaw). Further analysis was carried out using a Python script employing the following operations: Savitzky-Golay filtering with factors 11 and 2 (only for component analysis), background removal after Zhang et al.,^41^ and normalization of data to the range of 0 to 1. The components were detected by non-negative matrix factorization algorithm using 10 components. The individual components were assigned to LCO, LLZO:Al,Ta, and further phases (mainly La_2_Li_0.5_Co_0.5_O_4_), but only LCO and LLZO:Al,Ta were relevant for subsequent analysis. The total loadings of the phases were calculated by addition of the single component loadings assigned to the phases at each measurement point. For all points that were either assigned to be formed mainly by LCO or LLZO:Al,Ta, the unfiltered data (only background-removed and normalized) was used to calculate the peak position of the LCO peaks. Due to the transparency of LLZO:Al,Ta at the used wavelength, the LCO/LLZO:Al,Ta interface could be characterized by the LCO peaks appearing in LLZO:Al,Ta spectra. Fitting was carried out with a pseudo-Voigt function. For each fit, the regression coefficient (*R*^2^) was calculated, and only fits with *R*^2^ > 0.85 were considered for further analysis. The calculated peak position was analyzed separately for both phases.

### Secondary Ion Mass Spectrometry

The SIMS samples were polished with SiC sandpaper up to #4000 followed by water free diamond suspension (3 μm). The instrument used for chemical mapping is a Thermo Fisher Scios DualBeam (focused ion beam–scanning electron microscope, FIB-SEM) equipped with an in-house developed mass spectrometer. The FIB consists of a monoisotopic gallium liquid metal ion source to generate ^69^Ga^+^. The SIMS system is based on a double-focusing magnetic sector and allows parallel detection of multiple masses. Other details of the instrument can be found elsewhere.^42^

SIMS images are recorded with an accelerating energy of the primary ions of 30 keV and currents between 0.3 and 0.5 nA. The sample stage is biased to a potential of +500 V, resulting in a primary ion impact energy of 29.5 keV. The measurements are performed in positive mode, and the detected masses are ^27^Al^+^, ^59^Co^+^, and ^155^LaO^+^. The image resolution is 512 × 512 pixels, and the dwell times per pixel are between 750 and 1000 μs. Data analysis was performed using the free software Fiji (ImageJ).^43^

### Thermodynamic Modeling and Raman Spectra Simulation by DFT

The details of the thermodynamic modeling by DFT are provided in the Supporting Information.

## Results

**Figure 1:**
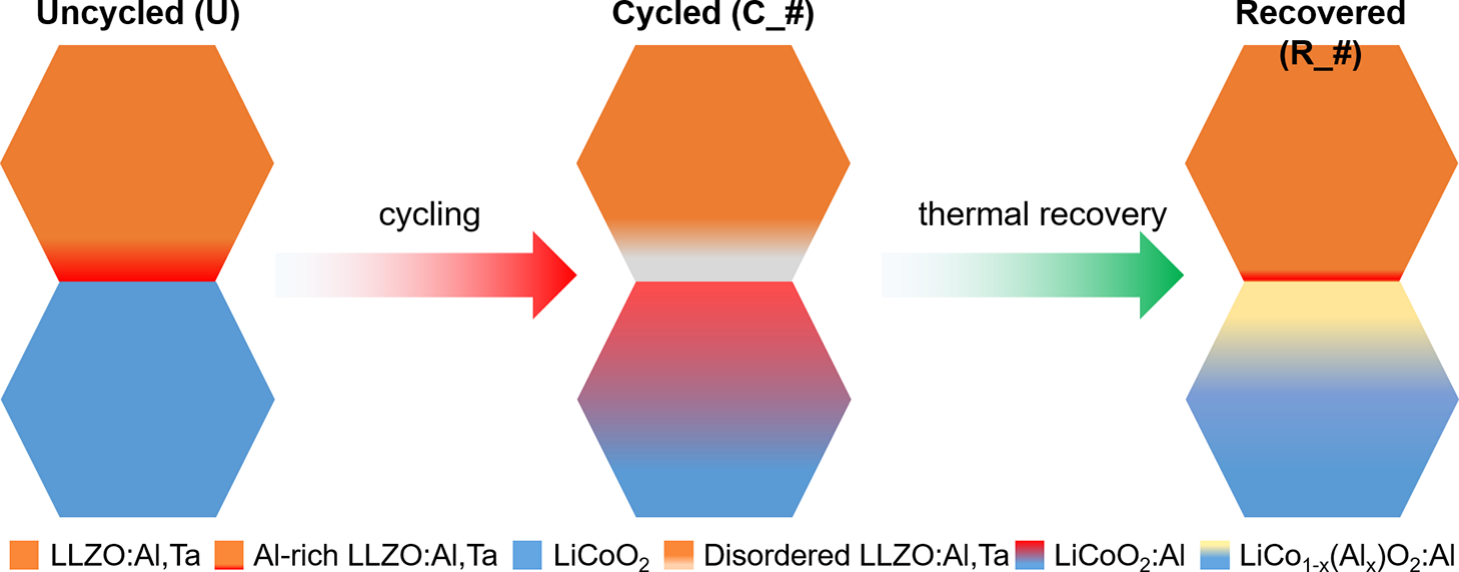
Overview scheme of the different states of the composite with a focus on their interface cathodes.

In our previous study, we demonstrated the use of the FAST/SPS sintering process for fabrication of all-ceramic half-cells consisting of a dense LLZO:Al,Ta separator and a composite LCO-LLZO:Al,Ta cathode.^17,32^ Due to the high applied mechanical pressure during sintering, FAST/SPS generally allows a significant reduction in the temperature and time required to fully densify and sinter the powders. Indeed, very dense cathodes (95% relative density) were obtained after FAST/SPS sintering for only 10 min at 675 °C at 440 MPa^17^ compared to over 1000 °C for several hours in conventional sintering.^21,44,45^ However, these apparently perfect cells with chemically “clean” interfaces and no detectable amounts of secondary phases exhibited poor electrochemical activity, which was attributed to their very high total impedance. Structural investigations showed that ion interdiffusion was suppressed at the LCO/LLZO:Al,Ta interface as the LLZO:Al,Ta was disordered or even amorphous at the LCO/LLZO:Al,Ta interface after FAST/SPS, causing high cell impedance. Therefore, an additional annealing step was performed at 1050 °C for 30 min in air to crystallize the amorphous LLZO:Al,Ta phase, which reduced the impedance and led to the exceptionally high capacities reported earlier.^17^ Since this is the starting point of the study presented here, the cells at this stage will be referred to as “U” for uncycled. The cell is then electrochemically cycled for a specified number of cycles, denoted as C_# for the respective cycle number #, and then treated with the proposed thermal recovery (annealing) method and cycled again, denoted as R_# for the cycle number # after thermal recovery (annealing). A schematic overview of the different states of the composite cathodes in the cell, with a focus on the LCO/LLZO:Al,Ta interface, is shown in Figure 1.

**Figure 2:**
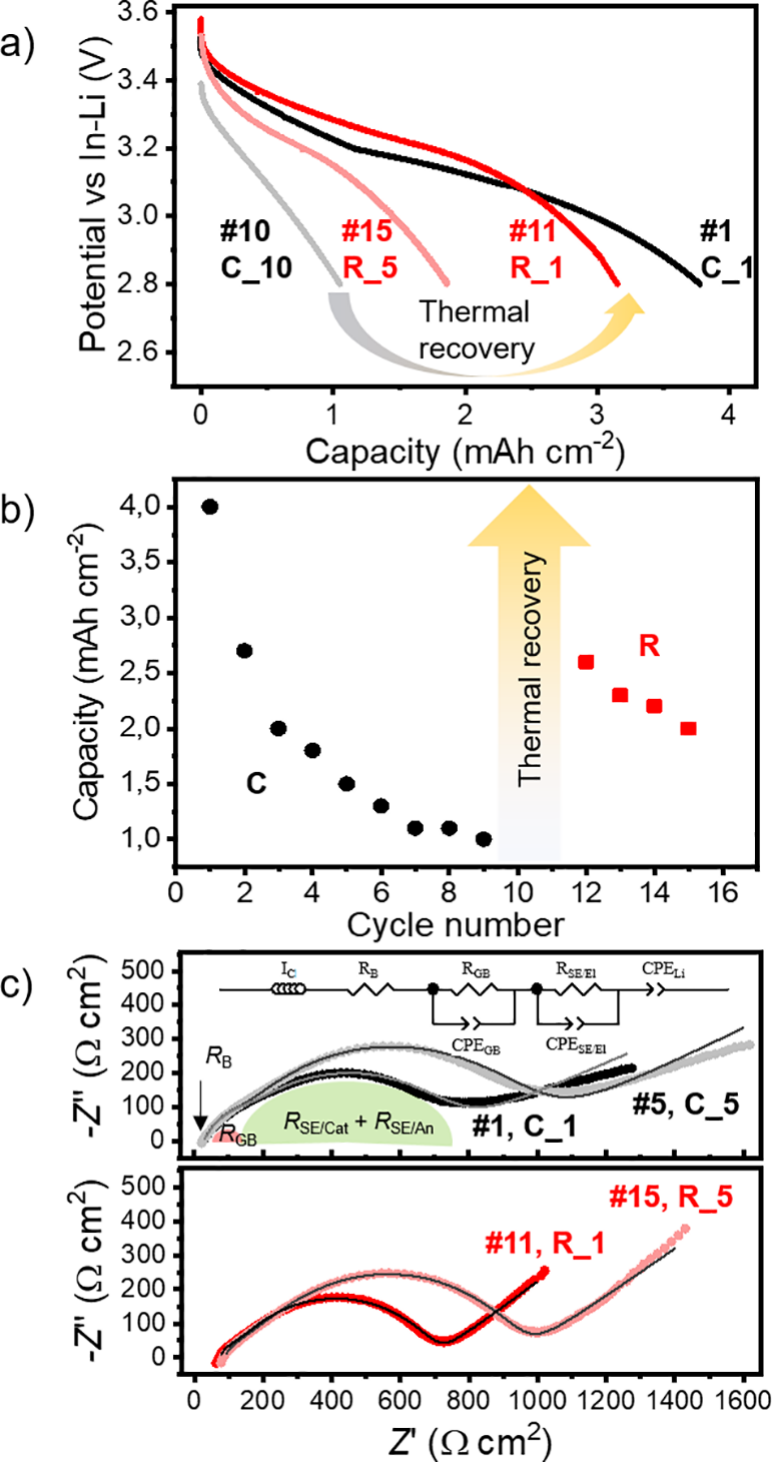
(a) Discharge curves of an ASSLB with a composite LCO-LLZO:Al,Ta cathode. The capacity of the ASSLB decays rapidly, but thermal recovery (annealing) restores most of the initial capacity. The capacities of each cycle are shown in panel b. (c) Nyquist plots of the EIS spectra recorded after performing the galvanostatic discharge curves in panel a. The equivalent circuit for the fitting is shown as inset in c. The impedance contributions of LLZO:Al,Ta bulk (*R*_B_) and grain boundary (*R*_GB_), solid electrolyte with the electrodes (*R*_SE/El_ = *R*_SE/cathode_ + *R*_SE/anode_), and Li-ion diffusion are exemplarily shown for the spectrum after the first discharge. In addition, the cable inductance (*I*_C_) was considered. The fitting parameters are given in Table S1.

Initially, the annealed/uncycled cells exhibit good electrochemical activity and a high areal capacity of about 4 mAh cm^–2^ in the first cycle (Figure 2a,b, C_1). However, a rapid decrease in capacity is observed with repeated cycling, with only 1 mAh cm^–2^ remaining after 10 cycles (Figure 2a,b, C_10). The EIS measurements of the cells (Figure 2c and Table S1) show that the areal resistance of LLZO:Al,Ta practically does not change during cycling, as it is 22 Ω cm^2^ for the annealed cell after the first charge (C_1) and 23 Ω cm^2^ after the fifth charge (C_5, details of the EIS analysis can be found in the Supporting Information). In contrast, the areal resistance of the grain boundary increases from 58 Ω cm^2^ after the first charge to 70 Ω cm^2^ after the fifth. However, the largest change is observed in the areal resistance of the interfaces between the electrode (cathode and anode) and LLZO:Al,Ta, which increases from 607 to 836 Ω cm^2^ within five cycles. In our previous work, a comparable system was analyzed by EIS. We found that after 5 cycles the impedance of LCO/LLZO:Al,Ta interface increased from 1081 to 1854 Ω cm^2^, whereas the impedance of the In–Li/LLZO:Al,Ta interface remained stable or slightly decreased from 25 to 15 Ω cm^2^.^32^ Moreover, the areal impedance of the In–Li/LLZO:Al,Ta interface is generally an order of magnitude smaller than that of the LCO/LLZO interface and less significant.^16^ The large increase in LCO/LLZO:Al,Ta impedance has been associated with amorphization of LLZO:Al,Ta due to cation diffusion between LCO and LLZO:Al,Ta, especially at high states of charge (SoC). Previous publications by Park et al.^46^ and our own study have shown that LCO loses Co and takes up Al during cycling, whereas LLZO loses Al and takes up Co, leading to amorphization of LLZO near the interface and partial inactivation of LCO.^32^ Because the ionic conductivity of LLZO is particularly sensitive to change in the crystal structure, this leads to the observed increase in interfacial resistance. If amorphization of the LLZO:Al,Ta during cycling is mainly responsible for the degradation of the cell performance, we postulated that the performance can be restored if LLZO:Al,Ta is recrystallized, e.g., with the same annealing procedure used for crystallization of the initial FAST/SPS-sintered cells. The advantage of this annealing approach is that it is similar to the original fabrication process. Since we used only inorganic materials, the annealing of the cycled cells can be done at the component level without having to completely disassemble the cell down to the raw material level. Therefore, in this study, the cycled full cell was disassembled, and only the current collectors and the In–Li anode were removed. The resulting half-cell (composite LCO-LLZO:Al,Ta cathode plus LLZO:Al,Ta separator) was then thermally recovered (annealed) under the same conditions as it was originally fabricated (see Materials and Methods for details). The thermally recovered (annealed) half-cell was then reassembled into a full cell and electrochemically analyzed (labeled “R_#”).

The first discharge after thermal recovery reached 3.1 mAh cm^–2^, which means that about 80% of the initial capacity of the composite LCO-LLZO:Al,Ta cathode or more than 70% of the lost capacity is recovered (Figure 2b). The slightly lower capacity after thermal recovery (annealing) may be due to incomplete reversal of the processes that led to the capacity fading or to the polishing off parts of the LCO-LLZO cathode after thermal recovery (annealing). In subsequent cycles, a similar capacity fading is observed as in the freshly annealed composite LCO-LLZO:Al,Ta cathode, but the degradation is slower and about half of the capacity is retained after 5 cycles, compared to only about 40% for the initial cell (Figure 2b). This observed recovery of the initial capacity after thermal recovery (annealing) is caused by the decrease in the total impedance of the cell (Figure 2c, R_1), which becomes very similar to the impedance of the initial cell (Figure 2c, C_1). However, in the following cycles, the semicircle in the midfrequency range increases again, similar to the freshly annealed cells, and ends again at higher values (Figure 2c, R_5). Fitting the impedance spectra shows that the bulk impedance of the LLZO:Al,Ta is practically unaffected by thermal recovery and cycling and remains constant at 24 Ω cm^2^. The grain boundary resistance, on the other hand, increases significantly after the thermal recovery (annealing) and reaches a value of 120 Ω cm^2^ (compared to 58 Ω cm^2^ for the freshly annealed cells, C_1) and increases slightly to a value of 135 Ω cm^2^ during the subsequent cycling (R_5), showing the same behavior as for the freshly annealed cells. The increased grain boundary resistance compared to the initial one indicates that the electrochemical performance recovers after thermal recovery (annealing), but the initial grain boundary structure of the LLZO:Al,Ta may not be restored. This might be due to the loss of Li within LLZO:Al,Ta or a result of secondary phase formation during annealing, as observed by Inada et al. during thermal recovery of LLZO:Ta electrolyte penetrated by Li dendrites.^47^

The largest change is observed in the impedances of the anode/LLZO:Al,Ta and the cathode/LLZO:Al,Ta interfaces, which are significantly reduced after the thermal recovery and are even slightly lower than before cycling. We suggested that this effect is due to the structural changes at the LCO/LLZO:Al,Ta interface and therefore analyzed the interface structure in detail in the following section of the paper. The change in the LCO/LLZO:Al,Ta interface impedance during cycling is similar to the initial cell, increasing from 553 to 808 Ω cm^2^ between the first and the fifth cycles.

**Figure 3:**
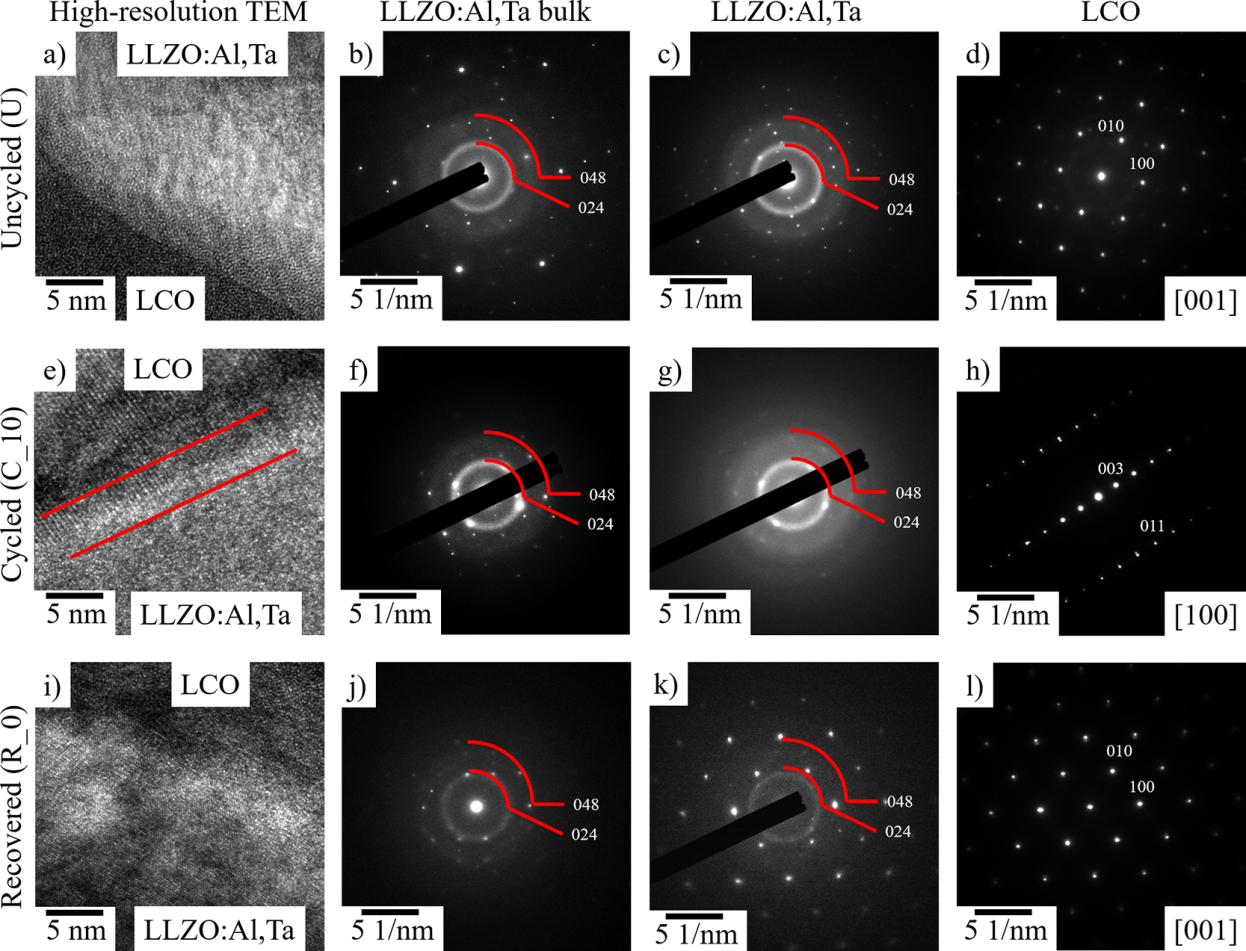
High-resolution TEM and SAED patterns of an uncycled (a–d), cycled (10 cycles, e–h), and recovered (i–l) LCO/LLZO:Al,Ta interface. In panel e, the formed interfacial layer between LCO and LLZO:Al,Ta is marked by red lines. The SAED of LLZO:Al,Ta bulk and LCO are measured around 1 μm away from the interface while LLZO:Al,Ta is measured at the LCO/LLZO:Ta,Al interface. The locations of the images and patterns are found in Figure S4. Panels a, c, d, and e are reprinted with permission from ref (32). Copyright 2022 American Chemical Society.

In order to obtain a comprehensive understanding of the degradation and thermal recovery mechanisms, as well as the processes that occur during electrochemical cycling and heat treatment (annealing), it is important to analyze the composite LCO-LLZO:Al,Ta cathode and its interface in detail after each treatment step. As observed in our previous work, the degradation was pinpointed to structural changes in the interface region, mainly to the amorphization of LLZO:Al,Ta during cycling.^32^ Using TEM and TEM-SAED, we showed that the uncycled/annealed state had sharp LCO/LLZO:Ta,Al interfaces and clear diffraction patterns of LCO and LLZO:Al,Ta phases (Figure 3a–d). After cycling, the LCO/LLZO:Ta,Al interface appears different. A brighter contrast is observed on the LLZO:Al,Ta side of the LCO/LLZO:Ta,Al interface, and it looks as if an interface layer has formed (Figure 3e, marked with red lines). Associated with this observation is a less defined SAED pattern, indicating a loss of crystallinity at the LCO/LLZO:Ta,Al interface (Figure 3b,c,f,g), while the LLZO:Al,Ta and LCO areas (about 1 μm away from the interface) appear unchanged (Figure 3d,h). However, some satellite peaks are observed in the LCO, indicating a slight change in the LCO structure (Figure 3h).

After thermal recovery (annealing) of our cell, TEM and TEM-SAED were performed again at similar locations (Figure S4). The high-resolution TEM image still shows a brighter contrast within the LLZO:Al,Ta in some areas, indicating that an interfacial layer between LCO and LLZO:Al,Ta remains (Figure 3i). The same distinct spots and rings are observed within the LLZO:Al,Ta grains (Figure 3j) as for the uncycled and cycled samples (Figure 3b,f,j), indicating that no obvious structural changes occur in the LLZO:Ta,Al bulk during cycling and thermal recovery (annealing). At the LCO/LLZO:Ta,Al interface, a much more distinct SAED pattern can be seen after thermal recovery, but a slight blurring remains, indicating that crystallinity may not yet be fully established (Figure 3k). In the LCO, on the other hand, the satellite peaks have now disappeared and a pristine, rhombohedral crystal structure can be observed also at the LCO/LLZO:Ta,Al interface (Figure 3l).

Although it is difficult to obtain atomic resolution for LLZO:Al,Ta in TEM due to its beam sensitivity, the images (Figure 3a,e,i) show evidence of a chemical change in the LCO/LLZO:Ta,Al interface during thermal recovery (annealing, Figure 3g,h,k,l). After cycling, the grain boundary is detected by a contrast change in the TEM image, and a slightly inhomogeneous contrast within the LLZO:Al,Ta at the grain boundary can be seen at high resolution (Figure 3a,e,i). This contrast change within the LLZO:Al,Ta is due to cycling, as it is not observed in the uncycled state (Figure 3a,e,g).^17,32^ However, after thermal recovery (annealing), the grain boundary appears in a brighter contrast than the LCO or LLZO:Al,Ta (Figure 3a,i). Whether this lighter contrast at the grain boundary is related to the LCO, LLZO:Al,Ta, or both is not entirely clear. In any case, it should be due to a change in the atomic number causing the altered contrast (*Z*-dependence). A change in average atomic number can only be caused by element diffusion, which itself must have a driving force. During cycling, the driving force can only be a difference in electrochemical potential; during heat treatment for recovery, it can only be the chemical potential between the most stable phases. To noticeably reduce the average atomic number, only diffusion of mobile elements makes sense. Besides Li, only Al is mobile enough to account for the observed changes. In-depth chemical and structural analyses are therefore required to investigate the processes that occur during cycling and thermal recovery (annealing).

**Figure 4:**
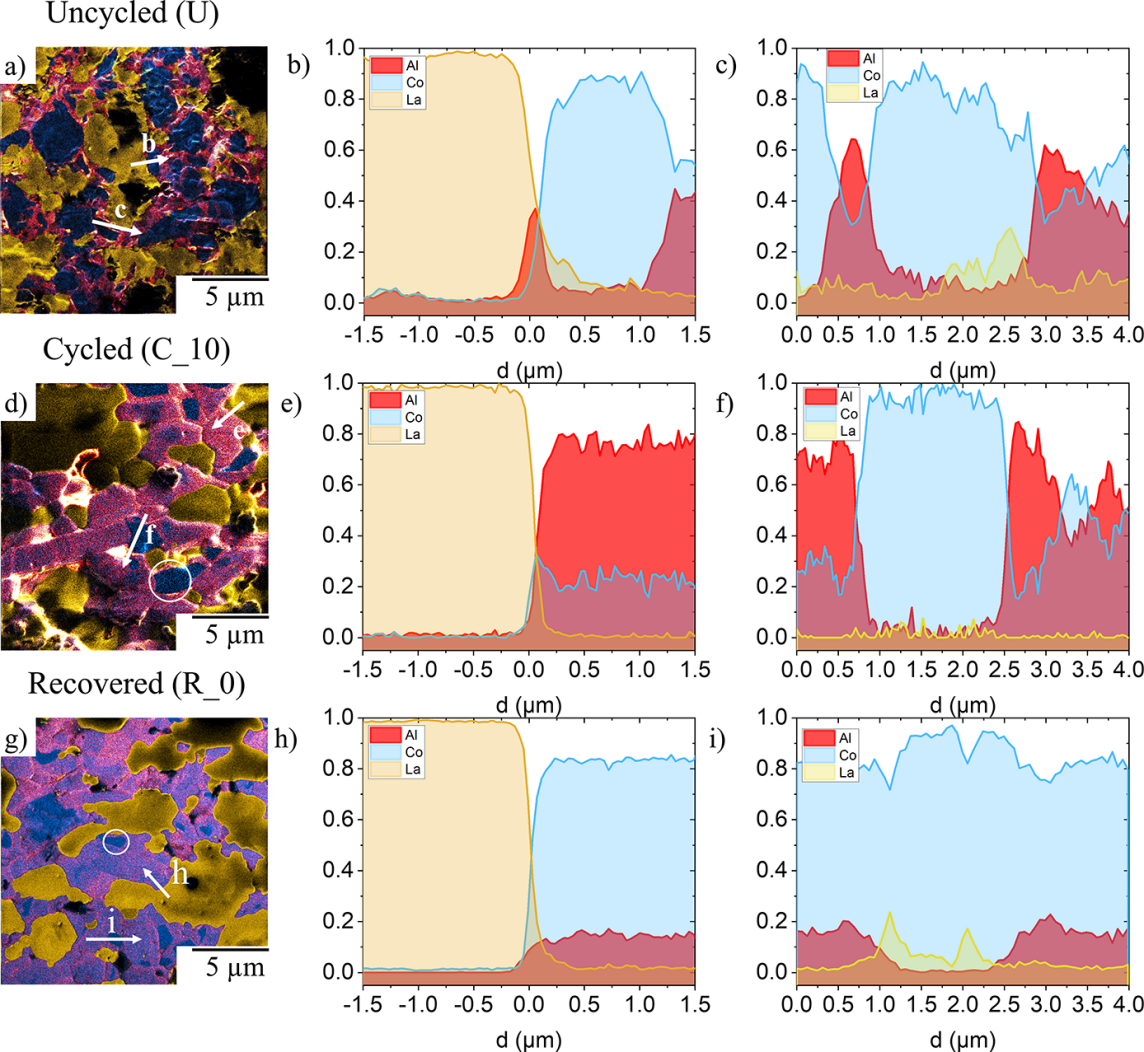
Elemental mappings with SIMS analysis for La (yellow), Al (red), and Co (blue) for the (a) uncycled, (d) cycled (10 cycles), and (g) recovered state. (The single elemental mappings are found in Figure S3.) Across the LCO/LLZO:Al,Ta interface (b, e, and h) and across the LCO/LCO interface (c, f, and i), SIMS line profiles have been prepared and the count for the La, Co, and Al signal is shown in relation to the combined elemental count in panels b and c for the uncycled, panels e and f for cycled, and panels h and i for the recovered state. The arrows in panels a, d, and g show the location and the direction of the SIMS line profiles.

To obtain information on the element redistribution at the microstructure level due to cation diffusion, high-resolution SIMS imaging was performed in all states: uncycled/annealed, cycled (10 cycles), and recovered. Figure 4 shows the combined elemental maps of La, Al, and Co obtained by SIMS for each of the three states of the composite LCO-LLZO:Al,Ta cathodes.

Qualitative assessment shows that Co and La are clearly separated initially (in the uncycled state, Figure 4a–c), being detected strictly within the grains of cathode active material (LCO) and the solid ion conductor (LLZO:Al,Ta), respectively. Interestingly, the Al signal is strongest at the LCO/LLZO:Al,Ta interfaces and between the LCO grains (Figure 4a–c). However, since the SIMS intensity is not directly related to concentration (matrix effect), quantification of the signal is difficult. The only conclusion that can be drawn here is that some interphases exist that have a matrix structure different from LCO or LLZO and contain Al. It is known from the sintering of Al-substituted LLZO that such interphases, often consist of lithium aluminates and promote sintering, e.g., by a liquid-phase sintering mechanism.^18^ It is therefore likely that such interphases also exist in the investigated composite LOC-LLZO:Al,Ta cathodes and foster their densification. For the cycled sample (Figure 4d–f), the Al distribution is different. Compared to the uncycled sample, some Al has diffused into the LCO, noticeable by a much broader distribution of the Al signal (red), but not uniformly, as some areas remain blue (Figure 4f). Since the LCO matrix is the same for the uncycled and cycled samples (both mappings were taken in the discharged state), the SIMS intensity is now a measure of the Al concentration in the LCO. Furthermore, the intensity for Al at the grain boundaries also appears to increase, but certainly not diminish. This suggests that Al-ions first diffuse from the LLZO:Al,Ta into the grain boundaries and subsequently into the LCO grains during electrochemical cycling. Since the cycling temperature is far below the sintering temperature at which thermally activated diffusion could occur, the only possible driving force for Al-ion diffusion is electrochemical potential. Although electrochemically activated Al-ion diffusion has been described previously in this material system,^46^ it has only been detected in a thin layer (a few hundred nanometers) near the interface. To our knowledge, this is the first time that Al-ion diffusion has been detected in the entire volume of the LCO in the range of several micrometers. Possible Co-ion diffusion from LCO into LLZO:Al,Ta was not observed by SIMS in any state, but it cannot be completely ruled out, either due to low Co ionization (low Co signal) within LLZO:Al,Ta or due to a diffusion length too small to be detected by SIMS (Figure 4). Interestingly, some Al-free LCO domains were observed within large LCO particles or within the center of LCO particle agglomerates (see white circle in Figure 4d), leading to the conclusion that these areas or grains are not electrochemically active, assuming that Al-ion diffusion is only electrochemically driven. Since LCO has much lower ionic conductivity compared to LLZO (and LLZO:Al,Ta), the interior of the larger grains might not be accessible for Li-ions in the given voltage range due to overpotentials associated with long-range diffusion within LCO. Some grains appear to be completely inactive, which can only be the case if the grain is somehow disconnected from the surrounding matrix, e.g., by a large amount of LCO around the particle, by cracks, or by poorly conducting secondary phases. Such a phenomenon has already been observed in other LCO-LLZO-based cathodes, where isolated LCO particles did not participate in cycling due to porosity or cracks.^48^ Since we can rule out porosity or cracks due to the high density of the samples, the presence of “inactive grains” in the composite LCO-LLZO:Al,Ta cathodes prepared by FAST/SPS can be attributed to the large volume fraction of LCO and the nonoptimized distribution of LCO and LLZO:Al,Ta particles. These “inactive LCO grains” could also explain why the cells did not reach 100% capacity during the first charging cycle. After thermal recovery (annealing, Figure 4g–i), the element distribution of the Al looks significantly different. The Al now seems to be more homogeneously distributed throughout the composite LCO-LLZO:Al,Ta cathode, especially in the LCO, even though some Al-free LCO grains remain (which again look like disconnected grains, white circle in Figure 4g). In addition, the Al signal no longer appears to be as pronounced at the grain boundaries, showing well-separated LCO/LLZO:Al,Ta interfaces. Since no electric field was involved during thermal recovery, this reorganization of Al occurred only by thermochemical diffusion in order to achieve a stable phase. Detailed modeling by DFT that can explain this observation is presented in the next section.

To examine the LCO/LLZO:Al,Ta interfaces in detail, line profiles for different positions on the images are shown in Figure 4. For the uncycled sample, Figure 4b,c shows a very low Al signal within LCO and LLZO:Al,Ta but a relatively high signal at the interface of LCO and LLZO:Al,Ta (Figure 4b). Although the Al concentration in LCO, LLZO:Al,Ta, and at the LCO/LLZO:Al,Ta interface cannot be quantified, the SIMS line profiles can be used for qualitative comparison of the element distributions after different treatment steps. After cycling, the Al signal inside LCO is much higher than before (Figure 4e), clearly indicating Al-ion diffusion in the LCO. For some grains, the Al signal remains low (Figure 4f), indicating that they did not participate in the electrochemical cycling. After thermal recovery (annealing), the Al signal is again lower, with rather sharp interfaces between LCO and LLZO:Al,Ta (Figure 4h,i). Note that the Al signal intensity is higher in LCO after thermal recovery (annealing) than in the freshly annealed sample (Figure 4b vs h). This change in signal intensity may be due to both a change in concentration and/or a change in matrix, i.e., the chemical environment surrounding Al.

Based on the SIMS analysis presented here, the presence of Al in LCO in the cycled and thermal recovered (annealed) samples is strong evidence that the electrochemically driven diffusion of Al-ions into LCO occurs during cycling and is not reversed by thermal recovery (annealing). This suggests that the amount of diffused Al-ions, the site they occupy in the LCO lattice, and the structure of the resulting LCO phases may be different at different treatment steps (Figure 4).

In order to estimate the possibility of different processes, the calculation of thermodynamically favorable sites for Al substitution was performed. In addition to thermal activation, it was considered that the thermodynamically favorable state could depend on the SoC of the LCO. In the charged state, half of the Li sites are vacant, which should favor the substitution of Al ions into the Li sites. In the discharged state, there are no Li vacancies, and the similar charge and ionic radii of Co^3+^ and Al^3+^ (both between 0.54 and 0.61 Å)^49^ should favor the Al substitution in the Co sites. To account for the different SoC, we considered the fully charged (Li_0.5_CoO_2_) and discharged (LiCoO_2_) LCO states for modeling. In addition, we considered the possibility of Al substitution at the octahedral or tetrahedral Li sites. The calculated formation energies for Al substitution at the Li or Co sites in the charged and discharged state are shown in Table 1. The formation energies were calculated as (details are presented in Tables S2 and S3)
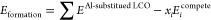


**Table 1:** Overview of the Calculated Formation Energies for Al-Substituted into LCO (Li_27_Co_27_O_54_) in the Li and Co Site in the Charged and Discharged State^a^

fully charged (Li_15_Co_27_O_54_**)** (eV/vacancy)			discharged (Li_27_Co_27_O_54_**)** (eV/vacancy)		
Al (octa. Li)	Al (tetra. Li)	Al (Co)	Al (octa. Li)	Al (tetra. Li)	Al (Co)
Li_15_AlCo_27_O_54_	Li_15_AlCo_27_O_54_	Li_15_Co_26_AlO_54_	Li_24_AlCo_27_O_54_	Li_24_AlCo_27_O_54_	Li_27_Co_26_AlO_54_
–0.9	7.08	0.03	11.37	14.75	3.64

a The detailed reactions are shown in Tables S2 and S3. octa., octahedral; tetra., tetrahedral.

In the charged state, the formation energy for Al-ions substituted at the octahedral Li site of LCO is negative. Therefore, Al-substituted Li_0.5_CoO_2_ is thermodynamically even more favorable than Al-free Li_0.5_CoO_2_. This fact explains the strong Al-ion diffusion observed in the electrochemically active regions during cycling as detected by SIMS (Figure 4) and agrees well with the calculated thermodynamically favorable Al(Li)-LCO phase. Since a positive formation energy was found for the other possible Al substitution sites, indicating thermodynamic instability of such substitution, Al should preferentially diffuse into charged LCO and occupy the octahedral Li sites. In contrast, for the discharged state of LCO, a positive formation energy is found for all possible Al substitution sites. Comparing the determined values on an absolute scale, the formation energy for Al substituting Co sites was found to be significantly lower than that for Li sites. However, Al-ion diffusion in the discharged LCO state during high-temperature sintering is frequently reported and experimentally confirmed.^9,44,46^ The driving forces here could be the entropy increase and the concentration gradient of Al between the Al-substituted and Al-free LCO. Based on the literature reports and the SIMS data (Figure 4g–i), we can conclude that although it is thermodynamically unfavorable, some Al substitution remains even in the discharged state of LCO after high-temperature heat treatment (thermal reovery, annealing) and could explain why the lost capacity could not be restored to 100% (Figure 2b).

**Figure 5:**
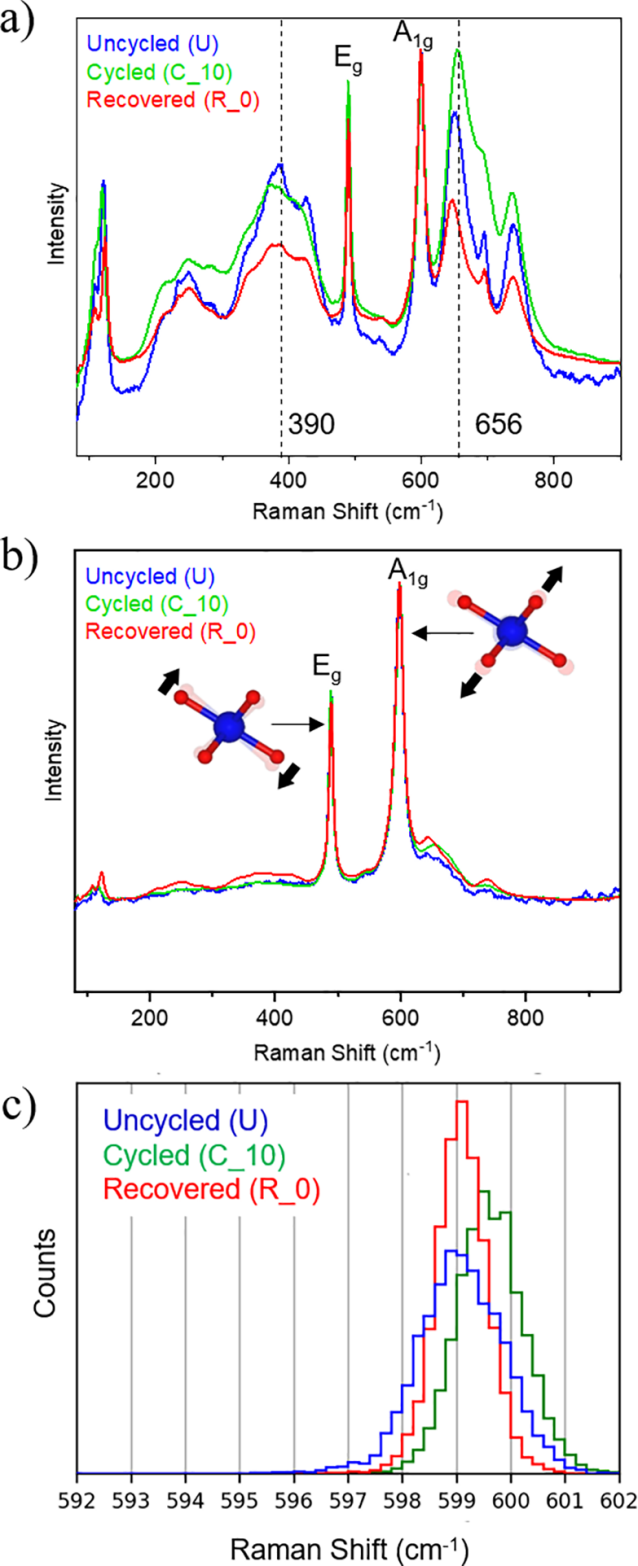
Averaged experimental Raman spectra of the composite LCO-LLZO:Al,Ta cathode for (a) LLZO:Al,Ta and (b) LCO in the uncycled, cycled (10 cycles), and thermally recovered (annealed) state. The positions of the A_1g_ peak of these composite LCO-LLZO:Al,Ta cathodes for each individual Raman spectrum from the Raman spectroscopy mapping are shown in panel c.

To obtain more information about the chemical environment of Al-ions and about the crystalline phases at the LCO/LLZO:Al,Ta interface, Raman spectroscopy mapping of the samples was performed (Figure 5). The Raman spectra of the LLZO:Al,Ta in the composite cathodes show the typical peaks of LLZO:Al,Ta and LCO (Figure 5a). The positions of LLZO:Al,Ta peaks are the same in all states (uncycled/annealed, cycled, and recovered) and are consistent with the expected peaks for LLZO.^44^ However, for the cycled sample, the peaks at 390 and 656 cm^–1^ are broadened (Figure 5a), consistent with the lower crystallinity of LLZO:Al,Ta observed in TEM (Figure 3).^32^ The Raman spectra of LCO after all treatment steps show the two active modes, E_g_ (around 489 cm^–1^) and A_1g_ (around 599 cm^–1^), of the rhombohedral LCO phase (Figure 5b). These values are slightly increased compared to the theoretically expected positions at 487 cm^–1^ (E_g_) and at 596 cm^–1^ (A_1g_).^50^ In all states, a small peak around 650 cm^–1^ is found, indicating the presence of Co_*x*_O_*y*_, which increases slightly with cycling and thermal recovery.^50^ To analyze the differences within the Raman spectra of LCO, a statistical approach was adopted (see Materials and Methods for details). For this analysis, the A_1g_ signal, which is due to Co–O stretching, was chosen because it has higher intensity and does not overlap with LLZO:Al,Ta peaks. Within the LCO spectra, the position of the peak maximum in each Raman spectrum was analyzed (Figure 5c). For an uncycled/annealed sample recorded at the LCO/LLZO:Al,Ta interface (Figure 5c), peaks are found between 596 and 601 cm^–1^, with most peaks at 599 cm^–1^. After cycling (C_10), the peaks are found between 598 and 602 cm^–1^. The peak distribution becomes narrower compared to the annealed state, but most peaks are found at 599 cm^–1^. Upon thermal recovery (annealing), the peaks shift reverses and peaks are observed between 597 and 601 cm^–1^. Most peaks are at 599 cm^–1^, and a higher number of peaks is found at this value compared to the other stages.

The shift in Raman peak position due to different lithiation states is well-known for LCO (Li_1–x_CoO_2_, x > 0); however, the peaks are typically red-shifted toward lower energies as the degree of lithiation decreases.^50^ The upward frequency shift is very unusual and requires a different explanation. As indicated by SIMS analysis (Figure 4), the Al-ions appear to be mobile during electrochemical cycling and migrate from the LLZO:Al,Ta to the LCO regions. Dobal and Katiyar performed a detailed Raman spectroscopy analysis of Al-substituted LCO and found that Al substitution in LCO at either the Li or Co site results in a blue shift of the E_g_ and A_1g_ peaks.^51^ Depending on the Al concentration (x ≤ 0.5 in Li_1−x_Al_1/3x_CoO_2_ or Li_1_Co_1−x_Al_x_O_2_), the A_1g_ peak can be shifted up to a wavenumber of 600 cm^–1^.

**Figure 6:**
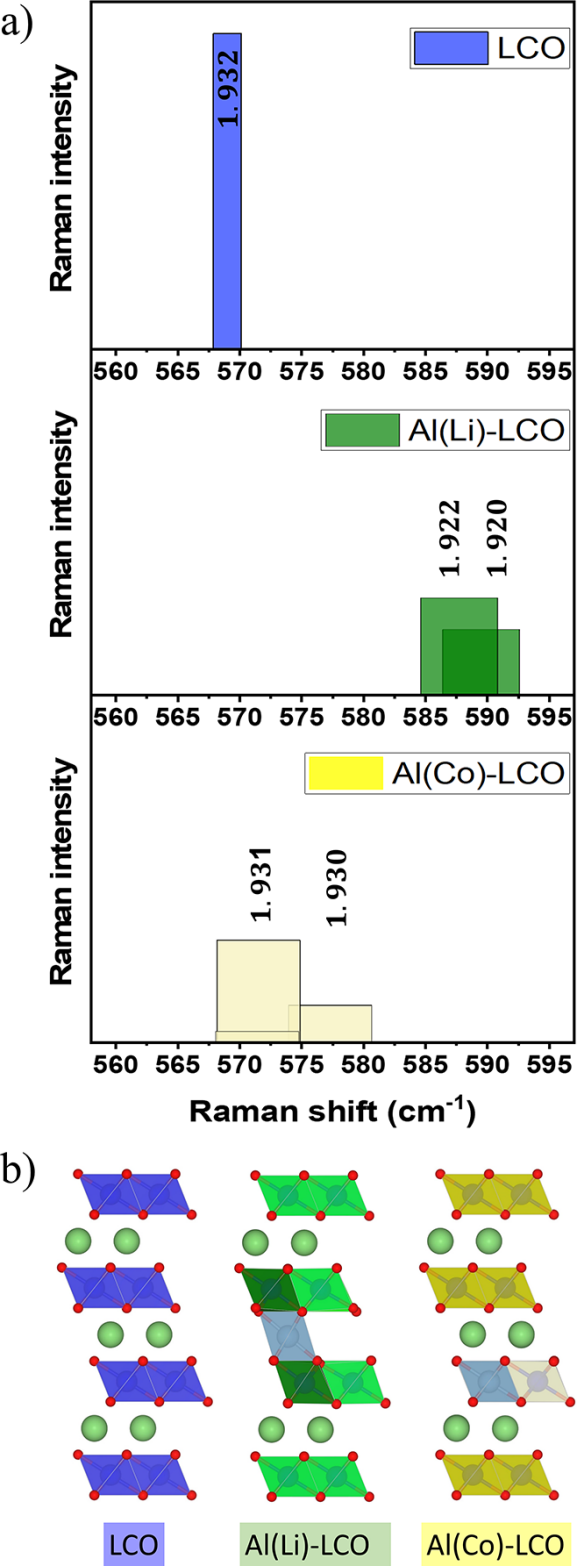
(a) Simulated Raman spectra of pristine LCO and Al substitution into LCO on the Li or Co site as well as (b) atomistic structure of the simulated LCO compounds (red: oxygen; dark blue, green, and yellow polyhedrals: Co; steel blue: Al; mint green: Li). To model Al-substituted LCO, a 2 × 2 × 1 unit cell (Li_12_Co_12_O_24_) with 8% Al concentration was used. With Al substituted into the Li site (Li_9_Co_11_Al_1_O_24_, Al(Li)-LCO), 3 Li vacancies were considered for charge balancing.

To investigate the effect of Al substitution on the position of LCO peaks, the Raman spectra of LCO without substitution and with Al substitution in the Li site (Li_9_Al_1_Co_12_O_24_, Al(Li)-LCO) and the Co sites (Li_12_Co_11_Al_1_O_24_, Al(Co)-LCO) were computed using DFT-Perdew–Burke–Ernzerhof (PBE) calculations (Figure 6). It should be noted that the PBE functional generally underestimates the vibrational frequencies as reported previously^52^ and provides lower **v** values (Raman shifts) compared to the measured peaks (436 and 569 cm^–1^, respectively) (Figure 5). Due to the errors in the approximated exchange-correlation functional (by PBE) and the arbitrary concentration of Al dopant, our calculations can only provide a qualitative understanding of the effect of substituting Al for Co or Li. Here, we focused on the Co–O stretching mode of the nearest-neighbor CoO_6_ octahedra to Al (hereafter referred to as *v*__A2g__). Our calculated Raman spectra show a blue shift for *v*__A2g__ for both substitution cases compared to pure LCO. The increase in *v*__A2g__ is significantly larger for the Al(Li)-LCO than that for the Al(Co)-LCO. To understand the peak shifts, the average Co–O bond lengths (*d*_Co–O_) along with the stretching mode, were calculated for these three structures. The average *d*_Co–O_ lengths for the nearest-neighbor CoO_6_ octahedra to Al in the Al(Co)-LCO system are slightly shorter than *d*_Co–O_ in bare LCO, which is why a small blue shift occurs in the former case. However, the *d*_Co–O_ lengths in Al(Li)-LCO are obviously shorter than in the other two cases, which is why the *v*__A2g__ for this system is the highest of the studied systems.

The experimental observation of the LCO peak at 599 cm^–1^ (Figure 5c) suggests that some Al-substituted LCO is already present in the uncycled state, especially at the LCO/LLZO:Al,Ta interface and between the LCO grains. This is plausible since SIMS analysis shows Al accumulation at the LCO/LLZO:Al,Ta interface (Figure 4a). During cycling, Al-ion diffusion into the charged (delithiated) LCO is thermodynamically favorable (Table 1), leading to increased Al substitution within the LCO and a more pronounced Raman peak shift. During thermal recovery (annealing), Al-ions could diffuse back into the LLZO:Al,Ta regions (if the Al concentration within LCO exceeds the one in LLZO:Al,Ta), leading to a smaller A_1g_ peak shift in the Raman spectra of LCO (Figure 5b,c). In addition, Al can shift its site occupancy within the LCO structure, e.g., into vacant Co sites. For example, Takahashi et al. calculated the effects of Al substitution on the LCO structure and found that Al occupying a Li site stresses the structure more than Al located on a Co position.^53^ If the Al changes its substitution site within the LCO, this would affect the Raman spectra by decreasing the peak shift and possibly also affect the SIMS analysis because the ionization energy could be different.

Based on the TEM and SIMS analysis, thermodynamic calculations, and Raman spectroscopy data, we propose the following degradation and recovery mechanisms:

### Diffusion of Al-Ions during Sintering

For Al-substituted LLZO, Al-ions are known to diffuse into grain boundaries during high-temperature exposure.^18^ In the case of a composite LCO and LLZO:Al,Ta system, Al ions appear to diffuse mainly into the LCO/LLZO:Al,Ta interface (and into the LCO–LCO grain boundaries, Figure 4). Diffusion is probably strongest at the LCO/LLZO:Al,Ta and LCO/LCO interfaces and is confined to a layer in the interface vicinity. Since the ionic conductivity of the resulting phase is presumably sufficient, substitution of Al in LCO does not seem to affect the electrochemical properties significantly.

### Electrochemically Driven Al-Ions Diffusion

Thermodynamic calculations suggest the favorable diffusion of Al-ions into charged (delithiated) LCO (Table 1). This diffusion may be very strong (as evidenced by SIMS, Figure 4). Since thermodynamic calculations indicate that this diffusion occurs along the Li sites, the exchanged Al would lead to blocking of the Li-ion diffusion pathways and thus to low ionic conductivity and electrochemical performance (Figure 2). At the same time, diffusion of Al from LLZO:Al,Ta leads to destabilization of the conductive cubic phase, resulting in the formation of disordered or amorphous LLZO:Al,Ta, which also exhibits low ionic conductivity (Figure 3). Therefore, both contributions lead to the observed increasing resistance (Figure 2c) and result in the capacity fade during cycling (Figure 2a,b).

### Reorganization of the Al Distribution during Thermal Recovery

Al substitution in LCO is thermodynamically unfavorable in the discharged state (Table 1), and therefore Al is “released” from LCO during heat treatment in the discharged state. However, based on the SIMS and Raman spectroscopy analysis, some Al-ions remain “trapped” in the LCO structure (Figures 4 and 5) even after thermal recovery (annealing). These Al ions most likely migrate from the Li site to a Co site within the LCO, resulting in less than 100% of initial capacity being recovered (Figure 2a,b). However, since the internal resistance of the cell is almost fully restored, both the blocking of Li-ion diffusion pathways by Al on Li sites inside LCO and the amorphization of LLZO:Al,Ta are reversed by the thermal treatment (Figures 5 and 3).

Our study shows that Al-ions readily diffuse during processing and cycling of the composite LCO-LLZO:Al,Ta cathodes, leading to significant changes in their structure, which could be the reason for the deterioration of cell performance. It remains to be investigated how other substituents, which are required to stabilize the cubic LLZO phase, affect the cathode performance. An important question is whether preventing Al-ion diffusion during processing and electrochemical operation could lead to garnet-based ASSLBs with stable cycling performance if, for example, Al-free LLZO is used. However, it should be noted that Al is the most economical dopant for practical application because it is cheap and essential for the sintering process and often appears as a processing-induced impurity.^16^ In this work, we show that Al-ion diffusion from LLZO:Al,Ta to LCO occurs during cycling and leads to a loss of crystallinity in LLZO:Al,Ta and potentially blocks Li-ion diffusion pathways in LCO. A thermal recovery (annealing) step at the component level recrystallizes the LLZO:Al,Ta and potentially leads to a change of the Al distribution in LCO, e.g., the change from the Li to the Co site. This opens the Li-ion pathways within LCO, so that cell performance can be restored very easily, even if Al-containing LLZO is used.

## Conclusions

We have shown that the electrochemical properties of an electrochemically degraded LCO/LLZO:Al,Ta interface can be recovered by thermal annealing, with restoration of high ionic conductivity and low interfacial resistance. The reason for that is the recrystallization of the electrochemically amorphized LCO/LLZO:Al,Ta interface.

Although the initial storage capacity is not fully restored, our method demonstrates the possibility of a cost-effective recycling of garnet (LCO-LLZO:Al,Ta)-based ASSLBs, without the need to decompose the cell components down to the raw material level.

In summary, by applying the method presented here, the recycling process of garnet (LCO-LLZO:Al,Ta)-basedASSLBs becomes simpler, more efficient, and thus more economical, with reduced environmental impact.

## Supporting Information Available

Descriptions of the DFT calculations for the thermodynamic and Raman spectroscopy analysis, microstructural analysis and EIS analysis, SEM images of the composite LCO-LLZO:Al,Ta cathode before and after cycling, SIMS mappings of the individual elements and their combination, TEM overview images, fitting values for the EIS measurements, and the values of the DFT calculations (PDF)
