# Supplementary material for: Thermal Recovery of the Electrochemically Degraded LiCoO2/Li7La3Zr2O12:Al,Ta Interface in an All-Solid-State Lithium Battery
Source: ACS Appl Mater Interfaces. 2023 Jan 17;15(3):4101–12. doi: 10.1021/acsami.2c20004 (PMC9881002; doi:10.1021/acsami.2c20004)
Supplement: Supplementary file 1 — am2c20004_si_001.pdf [file am2c20004_si_001.pdf]

Supporting Information

Thermal Recovery of the Electrochemically Degraded $LiCoO_2/Li_7La_3Zr_2O_{12}$:Al,Ta Interface in All-Solid-State Lithium Battery

Martin Ihrig[a], Liang-Yin Kuo[b], Sandra Lobe[a], Alexander M. Laptev[c], Che-an Lin[d], Chia-hao Tu[e], Ruijie Ye[a], Payam Kaghazchi[a,f], Luca Cressa[g], Santhana Eswara[g], Shih-kang Lin[d,e,h], Olivier Guillon[a,i], Dina Fattakhova-Rohlfing[a,j]*, and Martin Finsterbusch[a,]**

[a] Institute of Energy and Climate Research – Materials Synthesis and Processing, Forschungszentrum Jülich GmbH, 52425 Jülich, Germany

[b] Department of Chemical Engineering, Ming Chi University of Technology, No. 84, Gungjuan Rd., New Taipei City 24301, Taiwan

[c] Łukasiewicz Research Network – Poznan Institute of Technology, 6 Ewarysta Estkowskiego St., 61-755 Poznań, Poland

[d] Department of Materials Science and Engineering, National Cheng Kung University, No.1, University Rd., Tainan City 701, Taiwan

[e] Hierarchical Green-Energy Materials Research Center, National Cheng Kung University, No.1, University Rd., Tainan City 701, Taiwan

[f] MESA+ Institute for Nanotechnology, University of Twente, P. O. Box 217, Enschede 7500AE, The Netherlands

[g] Luxembourg Institute of Science and Technology, Advanced Instrumentation for Nano-Analytics (AINA), rue du Brill 41, 4422 Belvaux, Luxembourg

[h] Program on Smart and Sustainable Manufacturing, Academy of Innovative Semiconductor and Sustainable Manufacturing, National Cheng Kung University, Tainan City 701, Taiwan

[i] Jülich-Aachen Research Alliance: JARA-ENERGY, 52425 Jülich, Germany

[j] Faculty of Engineering and Center for Nanointegration Duisburg-Essen, University Duisburg-Essen, Lotharstr. 1, 47057 Duisburg, Germany

Corresponding Authors

Martin Ihrig – Institute of Energy and Climate Research – Materials Synthesis and Processing, Forschungszentrum Jülich GmbH, 52425 Jülich, Germany; Phone: +49-2461-6196841; E-mail: m.ihrig@fz-juelich.de; Fax: +49-2461-619120

Martin Finsterbusch – Institute of Energy and Climate Research – Materials Synthesis and Processing, Forschungszentrum Jülich GmbH, 52425 Jülich, Germany; E-mail: m.finsterbusch@fz-juelich.de

Dina Fattakhova-Rohlfing – Institute of Energy and Climate Research – Materials Synthesis and Processing, Forschungszentrum Jülich GmbH, 52425 Jülich, Germany; E-mail: d.fattakhova@fz-juelich.de

Experimental Section and Methods

Thermodynamic modelling by DFT

Atomic structures and spin density distribution were visualized with the VESTA program.[1]

Spin-polarized DFT calculations were performed using the Projector Augmented-Wave (PAW) pseudopotential method implemented in the Vienna Ab Initio Simulation Package (VASP) code.[2-3] The Perdew-Burke-Ernzerhof (PBE) functional was employed to approximate the exchange-correlation (XC) energy for all DFT calculations. Total Coulomb-energy (E_C) calculations were carried out using the supercell code.[4-5] DFT calculations were carried out using a Gamma-centered 2×2×1 k-point mesh for Li_xCoO_2 and 1×1×1 k-point mesh for $Li_xLa_3Al_yCo_zZr_{1.625}Ta_{0.375}O_{12}$. An energy cutoff of 600 eV as well as an energy and force convergence criterion of 10^{-4} eV and 10^{-2} eVÅ^{-1}, respectively, were used for all DFT calculations. To find the atomic structure of the cubic $Li_7La_3Zr_2O_{12}$ (c-LLZO) with Al and Ta dopants, namely $Li_{50}La_{24}Al_1Zr_{13}Ta_3O_{96}$, we started with the c-LLZO structure (i.e., $Li_{56}La_{24}Zr_{16}O_{96}$) from our previous work.[6] Since computing E_C values for all possible combinations of ($\frac{56!}{1!55!} \times \frac{55!}{50!5!} \times \frac{16!}{3!13!} = 56 \times 3478761 \times 560 = 1.1 \times 10^{11}$) is a formidable task, we performed two sets of calculations: 1) Finding position of 1 Al and 53 Li in 56 Li sites ($\frac{56!}{1!55!} \times \frac{55!}{53!2!} = 56 \times 1485 = 83160$ configurations) in $Li_{56}La_{24}Zr_{16}O_{96}$ and then fixing the position of Al and finding the positions of 3 Ta in 16 Zr sites and 50 Li in the determined 53 Li sites in $Li_{53}La_{24}Al_1Zr_{16}O_{96}$ ($\frac{16!}{3!13!} \times \frac{53!}{50!3!} = 560 \times 23426 = 1.3 \times 10^7$ configurations), 2)

Finding position of 3 Ta in 16 Zr sites and 53 Li ions in 56 sites in $Li_{56}La_{24}Zr_{16}O_{96}$ ($\frac{16!}{3!13!} \times \frac{56!}{53!3!} = 560 \times 27720 = 1.6 \times 10^7$ configurations) and then finding position of 1 Al in 53 Li sites and 50 Li in 52 Li sites in $Li_{53}La_{24}Zr_{13}Ta_3O_{96}$ ($\frac{53!}{1!52!} \times \frac{52!}{50!2!} = 53 \times 1326 = 70278$ configurations). It

was found that the determined structure from set 1) has the lowest E_C value, and it was, thus, considered for DFT-PBE calculations.

The formation energies of Al in different sites of charged and discharged LCO and the competing phases in the Li, Co, Al, O four elements system were obtained by performing first-principles calculation base on density functional theory (DFT).[7] The Projector Augmented-Wave (PAW)[2] pseudopotential implanted in Vienna *Ab Initio* Simulation Package (VASP)[3, 8] was used for spin-polarized DFT calculation. The Perdew-Burke-Ernzerhof (PBE)[4] functional was adapted for the exchange correlation. DFT+U method was used for energy modification in the calculations of transition metal oxides.[9] The U value for Co, which is 3.32 eV, was taken from Materials Project.[10] To further obtain more accurate formation energy calculation, an energy correction (1.87 eV) for calculating Co containing oxides formation energies was applied.[11-12] $Li_{27}Co_{27}O_{54}$ cell was built for discharged LCO, and $Li_{15}Co_{27}O_{54}$ model was used for charged LCO. To obtain Al stability at Li layer octahedral site, Li layer tetrahedral site, and Co site in discharged LCO, $Li_{24}AlCo_{27}O_{54}$ (Al in Li layer octahedral site), $Li_{24}AlCo_{27}O_{54}$ (Al in Li layer tetrahedral site, and $Li_{24}AlCo_{26}O_{54}$ models were built, respectively. On the other hand, $Li_{15}AlCo_{27}O_{54}$ (Al in Li layer octahedral site), $Li_{15}AlCo_{27}O_{54}$ (Al in Li layer tetrahedral site), and $Li_{15}AlCo_{26}O_{54}$ models were built for Al in different sites of charged LCO. The k-point mesh for Al-doped LCO calculation was 2×2×1, and all the calculations were done with the criterion of 10^{-4} eV energy convergence and 10^{-2} eV/Å force convergence.

Calculation of Raman spectra of LCO by DFT

Raman spectra of LCO as well as LCO with Al substitution into the Li- and Co-site were computed using DFT-PBE calculation (Fig. 6a,b). The centers of ν values computed by DFT-PBE for pure LCO are at 569 cm^{-1} and 436 cm^{-1} (Fig. 6a), which is 37 cm^{-1} and 44 cm^{-1} lower, respectively,

compared to the experimental values (Fig. S5). Underestimation of vibrational frequencies is, however, typical for the DFT-PBE calculation and has also been reported for other systems in previous theoretical studies using the PBE functional.[13] To model Al-doped LCO we used a 2×2×1 unit cell ($Li_{12}Co_{12}O_{24}$) in which the concentration of Al is 8%. Because of the errors in the approximated exchange-correlation functional (by PBE) and high concentration of Al dopant, only a qualitative understanding of impact of substitution of Co or Li by Al is expected from our calculation. Here, we focused on the large-intensity bar with a center at ν_c = 569 cm^{-1}, which is due to the Co−O stretching (see Fig. 6a). For Al substituted into the Li site ($Li_9Al_1Co_{12}O_{24}$:Al(Li)-LCO), 3 Li vacancies were considered for charge-balancing. The computed $\nu_{A_{2g}}$ values split to two parts which are higher than the bare LCO system With Al substituted into the Co site ($Li_{12}Co_{11}Al_1O_{24}$, Al(Co)-LCO), we replaced a Al with Co. The simulated Raman spectra for Al(Li)-LCO split into three parts. One is close to the major peak of pristine LCO ($\nu_c \approx$ 569 cm^{-1}), and the other two are $\nu_c \approx$ 577 cm^{-1} and 582 cm^{-1}. To understand the peak shift and split in simulated Raman spectra, the average Co−O bond lengths (d_{Co-O}) along which the stretching mode exists were calculated for these three structures (Fig. 5b). The CoO_6 octahedra were colored according to their d_{Co-O} lengths. Computed d_{Co-O} for all Co−O bond lengths in the pristine LCO is 1.932 Å. However, the computed $\nu_{A_{2g}}$ values for CoO_6 octahedra in Al(Li)-LCO that are nearest neighbor of Al cations are 588 cm^{-1} and 590 cm^{-1}. They are 19 cm^{-1} and 21 cm^{-1} higher than that of bare LCO. This is because the calculated d_{Co-O} for the former case (d_{Co-O} =1.920 Å and 1.922 Å) is shorter than that bare LCO (d_{Co-O} = 1.932 Å). The average d_{Co-O} length for most of CoO_6 octahedra in the Al(Co)-LCO system (d_{Co-O} = 1.921 Å) is shorter than that in the bare LCO. The d_{Co-O} values for CoO_6 octahedra in Al(Co)-LCO that are at the same layer as AlO_6 octahedra are, however,

larger (d_{Co-O} = 1.930 Å and 1.931Å) and closer to those in the bare system leading to the appearance of two small peaks with $\nu_{A_{2g}}$ = 577 cm^{-1} and 572 cm^{-1} close to $\nu_{A_{2g}}$ = 569 cm^{-1} in the bare LCO.

Microstructural analysis

The microstructure was investigated by Scanning Electron Microscopy (SEM) with a Zeiss Ultra 55 microscope in back-scattering mode at 15 kV. Samples were embedded in epoxy and polished or used as fracture surface analysis. First SiC sandpaper up to #4000 was used followed by water free diamond suspensions (9 µm, 3 µm, and 1 µm). The surface electronic conduction was increased by sputtering of a thin Pt- (EM ACE200, Leica) or Au-layer (Cressington 108).

Electrochemical impedance analysis

In the Nyquist plots (Fig. 2c) a semicircle in the high frequency range attributed to the total (bulk, b and grain boundary, gb) resistance of the solid electrolyte, a stretched semicircle in the mid- to low frequency range, and the Li-ion diffusion tail in the low frequency range is visible. The mid- to low frequency range is interpreted as a combination of the individual impedances of the anode and the cathode. The medium frequency contribution is assigned to the LCO/LLZO:Al,Ta impedance and the low frequency contribution to the LLZO:Al,Ta/In-Li impedance based on the observation made by Janek and coworkers,[14-15] Sakuda et al.[16] as well as in our previous analysis of the LCO/LLZO:Al,Ta system.[17] Often, also the capacitance values are used to assign the individual contributions.[15, 18-19] However, this approach requires the separation of the individual contributions within the EIS spectra. In our EIS spectra, only two capacitances are accessible. So, the contributions assigning based on the capacitance value is challenging (Table S1).

Figures

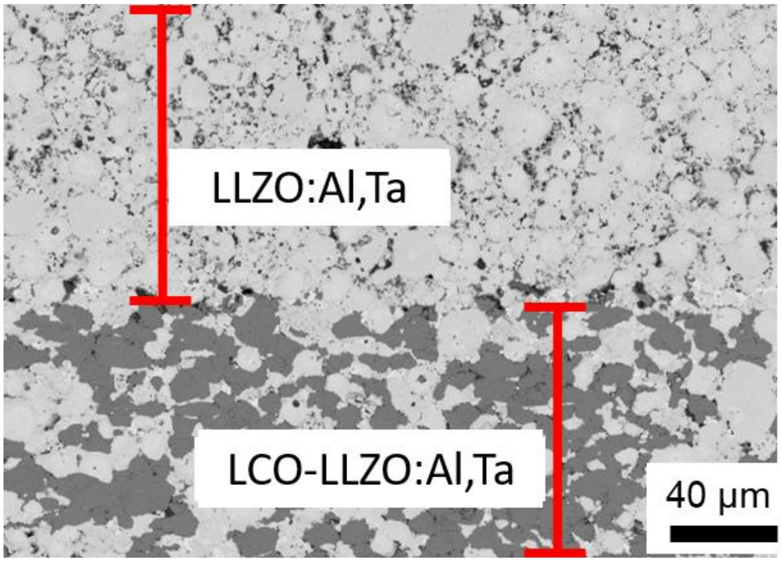

Figure S1. Cross sectional SEM image of the prepared half-cell by FAST/SPS at 750 °C with 440 MPa applied pressure in Ar atmosphere. The LCO-LLZO:Al,Ta layer has a density of around 95% while the LLZO:Al,Ta separator layer has a density of around 92%.

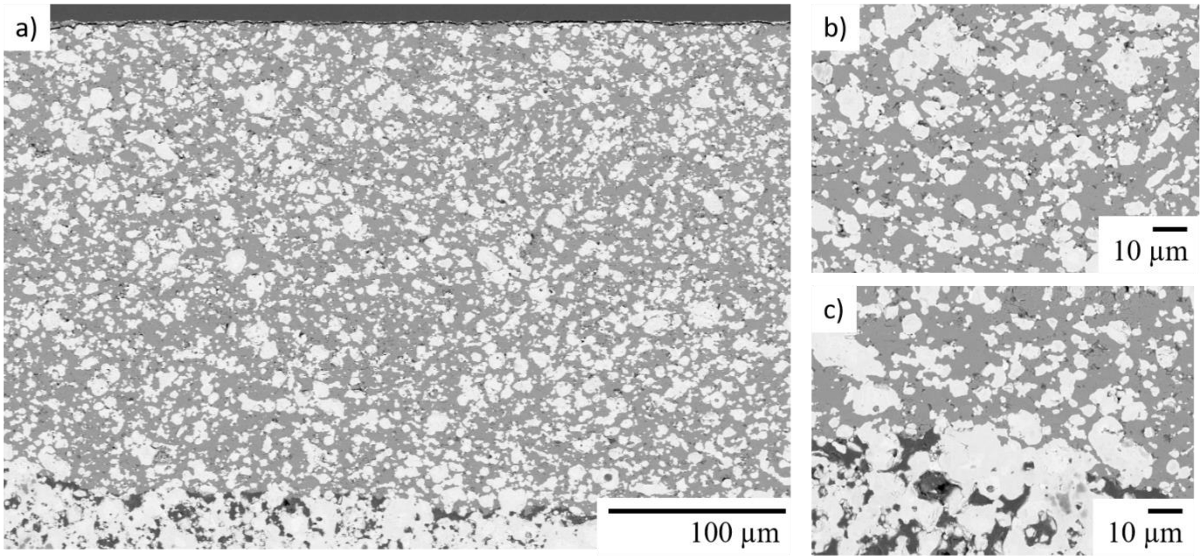

Figure S2. a) SEM cross sectional images of the composite cathode after electrochemical cycling. The LCO/LLZO:Al,Ta interface appears crack-free and intact. No obvious mechanical fracturing is observable. The image in b) is taken from the current collector side and the image in c) from the interface between LCO/LLZO:Al,Ta composite cathode and LLZO:Al,Ta separator.

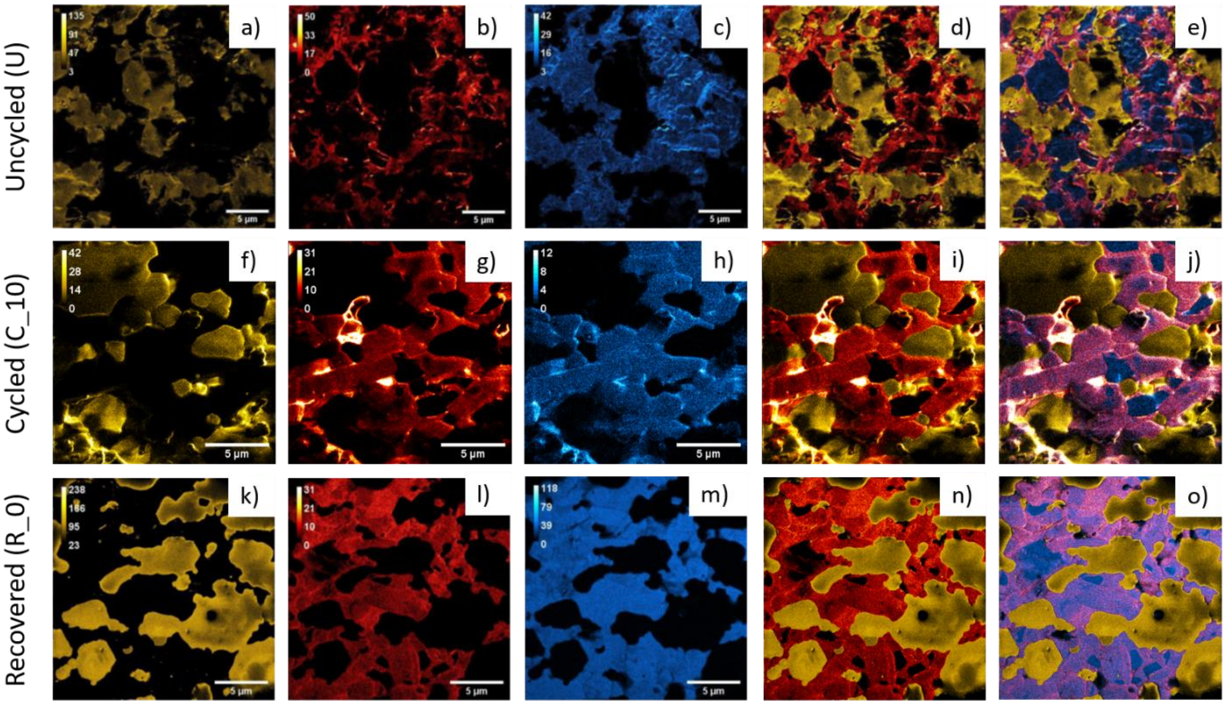

Figure S3. Elemental mappings via SIMS analysis for La (yellow), Al (red), Co (blue) and the overlays of La and Al (yellow and red) and for all elements (yellow, red, and blue). The SIMS mappings have been performed for the uncycled (a-e)-, cycled (f-j)-, and recovered-state (k-o).

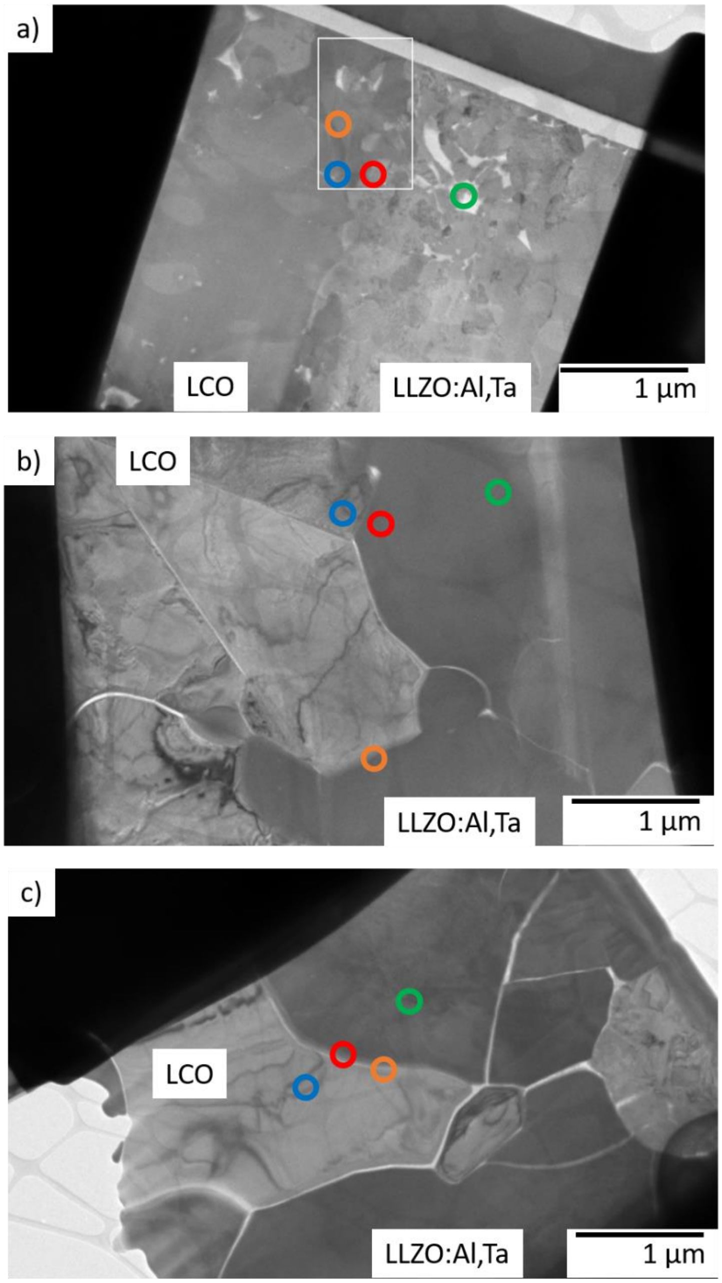

Figure S4. TEM images of an uncycled (a), cycled (b), and recovered (c) LCO/LLZO:Al,Ta interface. The circles show the locations of areas investigated by high-resolution TEM in orange and; blue for LCO; green for LLZO:Al,Ta; red for LLZO:Al,Ta bulk for the SAED pattern shown in Fig. 3. a) Reprinted with permission from ACS Appl. Mater. Interfaces 2022, 14, 11288. Copyright 2022 American Chemical Society.

Tables

Table S1. Fit results for ASSLB with a LCO-LLZO:Al,Ta cathode. R represents an ohmic resistor and CPE a constant phase element. The indices are: C: cable, B: bulk/grain, GB: grain boundary and Se/El: Solid electrolytes/electrode (cathode and anode). The exponent for the CPE is denoted as n. The capacity (*C*) was calculated with the following formula: $C = (CPE \cdot R)^{(1/n)} / R$.

Cycle No.	1 (C_1)	5 (C_5)	11 (R_1)	15 (R_5)
I_C	6,5E-11	1,3E-6	2,2E-6	3,4E-6
R_B	21,7	22,6	24,2	24,0
R_{GB}	58,8	69,7	119,6	135,4
CPE_{GB}	3,8E-7	3,3E-7	1,5E-6	1,3E-6
n_{GB}	0,8	0,8	0,6	0,6
C_{GB} (F)	2.6E-8	2.5E-8	1.7E-9	1.5E-9
$R_{SE/El}$	607,0	836,2	552,8	808,4
$CPE_{SE/El}$	1,8E-6	1,6E-6	1,5E-6	1,8E-6
$n_{SE/El}$	0,7	0,7	0,7	0,7
$C_{SE/El}$ (F)	6.2E-8	3.0E-8	8.E-8	3.5E-8
CPE_{Li}	6,5E-4	6.1E-4	2.9E-3	2.0E-3
n_{Li}	0,3	0,3	0,4	0,4

Table S2. Formation reaction in the charged state.

Al position	Al-substituted LCO	Initial compounds	Formation energy (eV/vacancy)
Al (octa. Li)	$Li_{15}AlCo_{27}O_{54}$	$Li_{15}Co_{27}O_{54}$ + Al	-0.9
Al (tetra. Li)	$Li_{15}AlCo_{27}O_{54}$	$Li_{15}Co_{27}O_{54}$ + Al	7.08
Al (Co)	$Li_{15}AlCo_{26}O_{54}$	$Li_{15}Co_{26}O_{54}$ + Al	0.03

Table S3. Formation reaction in the discharged state.

Al position	Al-substituted LCO	Initial compounds	Formation energy (eV/vacancy)
Al (octa. Li)	$Li_{24}AlCo_{27}O_{54}$	$Li_{24}Co_{27}O_{54}$ + Al	11.37
Al (tetra. Li)	$Li_{24}AlCo_{27}O_{54}$	$Li_{24}Co_{27}O_{54}$ + Al	14.75
Al (Co)	$Li_{27}AlCo_{26}O_{54}$	$Li_{27}Co_{26}O_{54}$ + Al	3.64

References

(1) Momma, K.; Izumi, F. VESTA 3 for three-dimensional visualization of crystal, volumetric and morphology data. *Journal of Applied Crystallography* **2011,** *44* (6), 1272-1276, DOI: doi:10.1107/S0021889811038970.

(2) Blöchl, P. E. Projector augmented-wave method. *Physical Review B* **1994,** *50* (24), 17953-17979, DOI: 10.1103/PhysRevB.50.17953.

(3) Kresse, G.; Furthmüller, J. Efficient iterative schemes for ab initio total-energy calculations using a plane-wave basis set. *Physical Review B* **1996,** *54* (16), 11169-11186, DOI: 10.1103/PhysRevB.54.11169.

(4) Perdew, J. P.; Burke, K.; Ernzerhof, M. Generalized Gradient Approximation Made Simple. *Physical Review Letters* **1996,** *77* (18), 3865-3868, DOI: 10.1103/PhysRevLett.77.3865.

(5) Okhotnikov, K.; Charpentier, T.; Cadars, S. Supercell program: a combinatorial structure-generation approach for the local-level modeling of atomic substitutions and partial occupancies in crystals. *Journal of Cheminformatics* **2016,** *8* (1), 17, DOI: 10.1186/s13321-016-0129-3.

(6) Moradabadi, A.; Kaghazchi, P. Effect of lattice and dopant–induced strain on the conductivity of solid electrolytes: application of the elastic dipole method. *Materialia* **2020,** *9*, 100607, DOI: https://doi.org/10.1016/j.mtla.2020.100607.

(7) Becke, A. D. Density-functional exchange-energy approximation with correct asymptotic behavior. *Physical Review A* **1988,** *38* (6), 3098-3100, DOI: 10.1103/PhysRevA.38.3098.

(8) Kresse, G.; Furthmüller, J. Efficiency of ab-initio total energy calculations for metals and semiconductors using a plane-wave basis set. *Computational Materials Science* **1996,** *6* (1), 15-50, DOI: https://doi.org/10.1016/0927-0256(96)00008-0.

(9) Wang, L.; Maxisch, T.; Ceder, G. Oxidation energies of transition metal oxides within the GGA+U framework. *Physical Review B* **2006,** *73* (19), DOI: 10.1103/PhysRevB.73.195107.

(10) Jain, A.; Ong, S. P.; Hautier, G.; Chen, W.; Richards, W. D.; Dacek, S.; Cholia, S.; Gunter, D.; Skinner, D.; Ceder, G.; Persson, K. A. Commentary: The Materials Project: A materials genome approach to accelerating materials innovation. *APL Materials* **2013,** *1* (1), 011002, DOI: 10.1063/1.4812323.

(11) Jain, A.; Hautier, G.; Ong, S. P.; Moore, C. J.; Fischer, C. C.; Persson, K. A.; Ceder, G. Formation enthalpies by mixing GGA and GGA+U calculations. *Physical Review B* **2011,** *84* (4), DOI: 10.1103/PhysRevB.84.045115.

(12) Lin, C.-a.; Nasara, R. N.; Lin, S.-k. Ab Initio Exploration of Co-Free Layered Oxides as Cathode Materials in Li Ion Batteries. *ACS Sustainable Chemistry & Engineering* **2021,** *9* (34), 11342-11350, DOI: 10.1021/acssuschemeng.1c02861.

(13) Miwa, K. Prediction of Raman spectra with DFT+*U* method. *Physical Review B* **2018,** *97* (7), 075143, DOI: 10.1103/PhysRevB.97.075143.

(14) Koerver, R.; Aygün, I.; Leichtweiß, T.; Dietrich, C.; Zhang, W.; Binder, J. O.; Hartmann, P.; Zeier, W. G.; Janek, J. Capacity Fade in Solid-State Batteries: Interphase Formation and Chemomechanical Processes in Nickel-Rich Layered Oxide Cathodes and Lithium

Thiophosphate Solid Electrolytes. *Chemistry of Materials* **2017,** *29* (13), 5574-5582, DOI: 10.1021/acs.chemmater.7b00931.

(15) Zhang, W.; Weber, D. A.; Weigand, H.; Arlt, T.; Manke, I.; Schröder, D.; Koerver, R.; Leichtweiss, T.; Hartmann, P.; Zeier, W. G.; Janek, J. Interfacial Processes and Influence of Composite Cathode Microstructure Controlling the Performance of All-Solid-State Lithium Batteries. *ACS Applied Materials & Interfaces* **2017,** *9* (21), 17835-17845, DOI: 10.1021/acsami.7b01137.

(16) Sakuda, A.; Hayashi, A.; Tatsumisago, M. Interfacial Observation between $LiCoO_2$ Electrode and $Li_2S-P_2S_5$ Solid Electrolytes of All-Solid-State Lithium Secondary Batteries Using Transmission Electron Microscopy. *Chemistry of Materials* **2010,** *22* (3), 949-956, DOI: 10.1021/cm901819c.

(17) Ihrig, M.; Finsterbusch, M.; Laptev, A. M.; Tu, C. H.; Tran, N. T. T.; Lin, C. A.; Kuo, L. Y.; Ye, R.; Sohn, Y. J.; Kaghazchi, P.; Lin, S. K.; Fattakhova-Rohlfing, D.; Guillon, O. Study of $LiCoO_2/Li_7La_3Zr_2O_{12}$:Ta Interface Degradation in All-Solid-State Lithium Batteries. *ACS Applied Materials & Interfaces* **2022,** *14* (9), 11288-11299, DOI: 10.1021/acsami.1c22246.

(18) Irvine, J. T. S.; Sinclair, D. C.; West, A. R. Electroceramics: Characterization by Impedance Spectroscopy. *Advanced Materials* **1990,** *2* (3), 132-138, DOI: 10.1002/adma.19900020304.

(19) Han, X.; Gong, Y.; Fu, K.; He, X.; Hitz, G. T.; Dai, J.; Pearse, A.; Liu, B.; Wang, H.; Rubloff, G.; Mo, Y.; Thangadurai, V.; Wachsman, E. D.; Hu, L. Negating interfacial impedance in garnet-based solid-state Li metal batteries. *Nature Materials* **2016,** *16*, 572, DOI: 10.1038/nmat4821
